# Can participatory video reduce mental illness stigma? Results from a Canadian action-research study of feasibility and impact

**DOI:** 10.1186/s12888-020-2429-4

**Published:** 2020-01-09

**Authors:** Rob Whitley, Kathleen C. Sitter, Gavin Adamson, Victoria Carmichael

**Affiliations:** 1 Department of Psychiatry, Douglas Hospital Research Centre, McGill University, 6875 LaSalle Blvd, Montreal, QC H4H 1R3 Canada; 2 University of Calgary, 2500 University Dr. NW, Calgary, AB T2N 1N4 Canada; 3 School of Journalism, Ryerson University, 350 Victoria St, Toronto, ON M5B 2K3 Canada

**Keywords:** Mental illness, Stigma, Recovery, Participatory video, Digital storytelling, Mixed-methods, Psychiatry, Canada, Feasibility, Participatory action research

## Abstract

**Background:**

Evidence suggests that stigma against people with mental illness remains high. This demands innovative approaches to reduce stigma. One innovative stigma reduction method is participatory video (PV), whereby marginalized people come together to script, film and produce bottom-up educational videos about shared issues. These videos are then shown to target groups. This paper has two objectives (i) to examine the feasibility of using participatory video with people with severe mental illness (SMI); and (ii) to assess viewer impressions of the resultant videos and subsequent subjective impact.

**Methods:**

We conducted a participatory action research study with three workgroups of people with severe mental illness situated in different Canadian cities, who set out to create and disseminate locally-grounded mental-health themed videos. This involved process and outcome evaluation to assess feasibility and impact. Specifically, we (i) observed fidelity to a co-designed action-plan in all three workgroups; (ii) distributed brief purpose-built questionnaires to viewers at organized screenings to assess preliminary impact; and (iii) conducted focus groups with viewers to elicit further impressions of the videos and subsequent subjective impact.

**Results:**

The three workgroups achieved high-fidelity to the action-plan. They successfully produced a total of 26 videos, over double the targeted number, during an 18-month period. Likewise, the workgroups organized 49 screenings at a range of venues attended by 1542 people, again exceeding the action-plan targets. Results from the viewer questionnaires (*N* = 1104, response rate 72%) indicated that viewers reported that their understandings had improved after watching the videos. Four themes emerged from six viewer focus groups (*N* = 30), with participants frequently noting that videos were (i) educational and informative; (ii) real and relatable; (iii) attention-grabbing; and (iv) change-inducing.

**Conclusions:**

To our knowledge, this study is the first large-scale multi-site project examining the feasibility and impact of a participatory video program for people with severe mental illness. The results indicate that participatory video is a feasible method in this population and gives preliminary evidence that resultant videos can reduce viewer stigma. Thus, participatory video should be considered a promising practice in the ongoing effort to reduce mental illness stigma.

## Background

Evidence suggest that community stigma against people with mental illness remains high across the world [1, 2]. Various studies indicate that public attitudes towards people with the most severe mental illnesses (SMI) such as schizophrenia remain particularly stigmatizing and inaccurate [3, 4]. Such attitudes have been witnessed in recent studies in Canada [5, 6].

For example, Stip et al. [7] surveyed over a thousand people in Quebec to discern attitudes towards schizophrenia. They found that 39% of respondents reported that schizophrenia provoked feelings of “suspiciousness”, while 54% said that people with schizophrenia are violent or dangerous. Data from a larger cross-Canada survey found that the majority of respondents would not visit a doctor (61%) or hire a lawyer (58%) with mental illness [8].

Other research indicates that such views are often held by important decision-makers including employers, journalists and even health care providers [5, 9, 10]. Such community stigma (also known as external stigma or public stigma) often leads to discrimination and exclusion for people with SMI and is a prominent obstacle towards recovery [6, 11, 12]. It can also lead to damaging self-stigma among people with mental illness [13].

Hence, stigma reduction has become a key target for mental health advocates in various jurisdictions across the world [11, 14]. In Canada, the most-recent ‘Mental Health Strategy for Canada’ states that national priority 1.1 must be to ‘increase awareness and reduce stigma’ [15]. As such, the Mental Health Commission of Canada (MHCC) created the ‘Opening Minds’ anti-stigma initiative, which has designed and implemented a comprehensive stigma reduction program, including the widespread use of contact-based sessions [16].

In these sessions, people with mental illness present their personal recovery journey to select audiences from a first-person perspective [17, 18]. Numerous studies indicate that these sessions can dispel negative stereotypes and improve attitudes towards people with mental illness [19–21]. Importantly, Corrigan [22] notes that these sessions have ‘the greatest impact when contact is targeted, local, credible and continuous … local priorities call for participatory action research (PAR)’. This approach propels the present study.

### Participatory video

Relatedly, health advocates have noted the potential of grassroots digital and visual methods to produce information and representations that can reduce stigma [23, 24]. One of these methods is known as *Participatory Video* (PV). In a PV project, a group of marginalized people come together to script, film and produce bottom-up educational videos about shared issues affecting them. The videos are rooted in a local context, and the workgroup has complete editorial control over content and themes [25].

Such videos can be based on a unifying topic in a documentary-style format or based on the experience of a single individual. This latter approach is known as *Digital Storytelling*, with an individual sharing ‘their story’ on camera, often focusing on overcoming challenges and strategies of resilience [26]. An integral part of the PV process involves organizing community screenings in the locality to educate viewers and catalyze change, as well as sharing resultant videos on-line via social media and other websites [23, 27].

These digital methods have much potential to reduce mental illness stigma, especially when local and targeted. Use of local people and places can root representations in familiar terrain. This can increase watchability, affinity and resonance when used with targeted local audiences [22]. This may be more effective than non-rooted video approaches which use celebrities or actors [5]. Moreover, PV can create a form of ‘mediated contact’, with the potential to confront stereotypes and illuminate day-to-day ‘behind the scenes’ realities of recovery. Indeed, one recent PV study found that videos produced by people with SMI were considerably more recovery-oriented and less focused on crime and violence than television news clips about mental illness [28].

However, recent reviews indicate an absence of studies examining whether PV videos produced by people with SMI can reduce mental illness stigma in viewers, and there is limited knowledge about feasibility and implementation [29, 30]. This is concerning given that much social science research indicates that we now live in a ‘digital age’ with increasing numbers of people obtaining information via on-line videos on mobile phones, tablets and home computers [31]. Mental health advocates and organizations could harness these innovative visual methods to reduce mental illness stigma should they prove effective; however, new efforts must be based on solid research and scientific evidence.

As such, we conducted a PV action-research study with people with SMI. The overall aim of this project was to form workgroups of people with SMI tasked to create and disseminate locally-grounded videos that could educate local viewers and reduce stigma. This involved simultaneous process and outcome evaluation to assess feasibility and impact. The present paper has two specific objectives: (i) to examine the feasibility of using PV with people with SMI, and (ii) to assess viewer impressions of videos and resultant subjective impact.

## Methods

The study began in 2015 and ended in 2019, with three sites. The study protocol was reviewed and approved by the Douglas Hospital Research Ethics Board (protocol 14/27). All participants were competent adults and gave written informed consent themselves. The research team consisted of the four study authors, as well as three local on-site videographer facilitators. To encourage buy-in and create a recognizable brand, we entitled the project and its activities *Recovery Advocacy Documentary Action Research* (RADAR). For ease of reading, the methods are divided into two sections related to the two specific objectives: (i) feasibility and (ii) impact.

### Feasibility

The successful grant application funding this study contained an action-plan which outlined four key sequential steps, namely (i) workgroup formation; (ii) classroom instruction; (iii) video production; and (iv) screening organization. This action-plan was co-developed by the research team and patient representatives from the three sites, thanks to a small Planning Grant. This allowed the first author to travel to five potential sites to present ideas and listen to perspectives. It also led to a two-day grant preparation workshop facilitated by two of the authors (RW & GA), attended by six patient representatives and three staff from the three eventual sites.

As such, the action-plan contained a series of targets and milestones (detailed below) which were co-developed by the research team and participants – an integral and desirable aspect of participatory action research [32, 33]. Importantly, this action-plan overlapped considerably with PV standard procedure, replicating approaches used in other successful PV projects [23, 25, 27].

First, we set out to create three workgroups of people with SMI located in three different Canadian cities (Montreal, Toronto, Halifax). For the purposes of this study, SMI was defined as an umbrella term covering schizophrenia, bi-polar disorder, major depression and schizoaffective disorder. These disorders share basic characteristics including high levels of community stigma and severe functional impairment when untreated [34]. Participants were recruited through snowball sampling from three psychosocial drop-in centres in these cities serving people with SMI through staff announcements, printed posters and word of mouth. We aimed to recruit between 6 and 10 core participants at each site who self-identified as in recovery from a SMI.

Second, we hired three professional videographer-facilitators (one per site) with teaching experience to conduct classroom instruction in video scripting, filming and editing. These classes were based on a pre-designed curriculum covering technical instruction in camera work and videography, as well as sessions on analytical thinking. This curriculum was designed by the study authors including a PV expert (KS), a stigma expert (RW) and a digital journalism expert (GA). We aimed to conduct twice-weekly training over a two-month period at the start of the project, followed by in-situ and booster training as needed for the rest of the project.

Third, the workgroups were tasked with creating a series of documentary-style videos about mental illness using their newly learnt skills, with assistance from the group videographer-facilitator. Each workgroup was given complete editorial control over the content, themes, segmentation and length of videos. That said, the action-plan stated that each workgroup would create at least four videos over an 18-month period (i.e. 12 videos overall).

Fourth, the workgroups were asked to organize a series of screenings in year three to three target groups: (i) tertiary students and young adults; (ii) health and social-service providers and (iii) the general public. These target groups were chosen somewhat opportunistically, as each site was well-connected with local educational and health care networks. However, this strategy overlapped with existing evidence, suggesting that both health-care providers [9] and young adults [17] often hold stigmatizing attitudes and inaccurate beliefs about SMI. The action-plan stated that each workgroup would organize a minimum of 12 screenings, 4 for each target group (i.e. 36 screenings overall), aiming for an average of around 20 people per screening. This means we aimed to reach 240 viewers per site (i.e. 720 overall).

The research team assessed fidelity to this action plan at each site to examine overall feasibility of a PV program for people with SMI through standardized observation of progress, comparing targeted outcomes with actual outcomes. This is consistent with best practice in feasibility research which demands that ‘feasibility outcomes’ are clearly pre-determined and described a priori; then objectively measured through replicable procedures [35].

To assess these outcomes, each videographer-facilitator wrote monthly reports detailing standardized information such as (i) training/ filming sessions given; (ii) number of attendees; (iii) activities undertaken; (iv) progress of videos; and (v) implementation barriers and challenges. Similarly, at least one member of the research team attended every screening, logging the number of attendees and distributing/ collecting evaluation questionnaires. Moreover, each workgroup electronically transferred each video to the first author at completion, which was logged and uploaded to a dedicated project website [36] and YouTube channel [37].

The monthly reports, progress logs and regularly updated field-notes were shared amongst the research team for discussion and clarification during monthly conference calls, which were minuted to add another record of progress. Achieving or exceeding the minimum targets described above by the end of the project were considered a sign of high-fidelity to the action-plan, indicating the feasibility of PV for people with SMI.

### Impact

Another objective was to examine viewer impressions of the resultant videos and subsequent subjective impact. This was assessed using both quantitative and qualitative measures which were co-designed by the research team and participants.

Quantitatively, we created a brief questionnaire to elicit basic information about the impact of the videos on viewers composed of five items. This was deliberately kept simple to maximise response rates and allow ease of comprehension and interpretation for the site participants. At the suggestion of the research team, two items were reverse coded to reduce bias:

1. The video changed my attitudes towards people with severe mental illness for the better
2. My understanding of stigma has not increased after watching this video
3. The video led me to better understand recovery from severe mental illness
4. The video had little impact on my outlook towards people with severe mental illness
5. My behaviour to people with severe mental illness will change for the better in light of this video.

Viewers rated the amount they agreed with each item using a 5-item Likert scale, ranging from “strongly disagree” to “strongly agree”, and were asked to include basic demographic information including age, sex and profession. Printed paper copies of these questionnaires were placed on seats before every screening, with announcements made asking viewers to complete them before they left. They were collected by the research team at the end of the screening and taken back to the office, where results were entered into Excel for storage. At the end of the project, responses were analyzed for measures of central tendency (mean, median, mode) as well as standard deviation and range.

At the end of the questionnaire, viewers were invited to leave an email address if they were interested in participating in a focus group to talk about their impressions of the videos and subjective impact. All those who left an email address were sent an email about times and days of upcoming focus groups and invited to RSVP and attend.

In total, we held six focus groups (two at each site) to explore in-depth perceptions of the videos, attended by 30 participants. The focus groups were facilitated by one of the authors, using a pre-designed topic guide with the recommended ‘funnel structure’ [38]. For example, early questions included ‘what did you think of the videos overall?’, followed by more specific questions such as ‘what did you think of the technical quality of the videos?’. Each focus group lasted 60 to 90 min in duration and were audio-recorded and transcribed verbatim. All focus group participants received $20 as compensation for their time.

The focus group data was analysed using a general inductive approach. This followed standard procedure of identifying and extracting common themes across transcripts [39]. Specifically, the first and last author read each transcript independently, identifying preliminary themes that captured common viewer impressions of videos and resultant subjective impact. These authors then met together to discuss their identified list of themes. Of note, both authors perceived a high degree of homogeneity within and between focus groups, meaning they identified a similar list of themes representing the underlying content. As such the authors quickly came to consensus over the most prominent themes, which were subsequently agreed upon as a faithful representation of the data by the whole research team.

## Results

In general, all three workgroups attained high-fidelity to the action-plan, achieving most of the listed goals, with some minor exceptions. Two workgroups achieved their core membership aims. The Montreal site had ten core members (six men & four women) and Halifax nine core members (five women & four men). The Toronto site had a core membership of four people (three men & one woman), slightly under the targeted number. Workgroup members ranged in age from 20 to 64 years of age. One site (Halifax) only contained members under 30, as the drop-in centre only served young people, while the other sites contained a broader mix of ages.

The classroom instruction was delivered with high-fidelity, with each site implementing the desired twice-weekly training early in the project over a two-month period. On average, nine people attended each session in Montreal, three people in Halifax and three people in Toronto. The numbers of people who regularly attended the classroom instruction are lower than the core workgroup membership, as some core group members joined the workgroups during the early stages of the video production phase, receiving in-situ training during this phase.

Importantly, each workgroup surpassed almost all feasibility targets for the number of videos produced, screenings organized, and viewers reached. As can be seen in Table 1 below, the three workgroups successfully produced a total of 26 videos, over double the targeted number, ranging in length from 2:20 min to 22:05 min (median length: 9 min 41 s).

**Table 1:** Target versus achieved number of videos, by site

	Halifax	Toronto	Montreal	All sites
Target	4	4	4	12
Achieved	7	10	9	26

Likewise, the workgroups generally exceeded targets in the organization of official screenings. In total, the workgroups organized 49 official screenings, exceeding the 36 targeted screenings as stated in the action-plan. Workgroups achieved or exceeded screening targets for students and for health/social service providers. However two sites failed to reach the number of targeted screenings for the general public. Of note, word of the project spread through the mental health service user communities in all three cities. This led to requests for screenings at mental health service user organizations and support groups during the dissemination phase. The workgroups agreed that such opportunistic screenings were both desirable and feasible. Thus, they organized a total of nine screenings to these groups, as detailed below in Table 2.

**Table 2:** Target versus achieved number of screenings for each target audience by site

Audience	Halifax		Montreal		Toronto		All sites	
	target	actual	target	actual	target	actual	target	actual
Students	4	4	4	7	4	4	12	15
General public	4	2	4	4	4	2	12	8
Health/social	4	4	4	6	4	7	12	17
Service-user	n/a	3	n/a	1	n/a	5	n/a	9
All audiences	12	13	12	18	12	18	36	49

The action plan stated that we would aim to screen the videos to a minimum of 720 viewers (240 per site). As can be seen in Table 3, the screenings were attended by a total of 1542 people, over double the targeted number. Importantly, the number of attendees within each target group was met or exceeded at each site. This included meeting targets for the general public, with the added bonus of 193 service-user viewers spread across the three sites.

**Table 3:** Target versus achieved number of attendees for each target audience, by site

Audience	Halifax		Montreal		Toronto		All sites	
	Target	actual	target	actual	target	actual	target	actual
Students	80	140	80	258	80	202	240	600
General public	80	80	80	92	80	137	240	309
Health/social	80	115	80	132	80	193	240	440
Service-user	n/a	82	n/a	20	n/a	91	n/a	193
All audiences	240	417	240	502	240	623	720	1542

Screenings generally lasted between 60 to 120 min and evolved over the course of the project with the latter screenings building on lessons learnt in the earlier screenings. This led to the construction of an organized screening model early in the dissemination phase, which was generally followed in latter screenings. This model included (i) an introduction by workgroup members; (ii) screenings of around 3–6 videos and (iii) a post-screening question period with panel discussion, including workgroup members. As such, screenings combined contact-based interaction with the information and stories contained in the videos.

### Impact

As mentioned, a total of 1542 people attended the screenings. Among these, 1104 completed the brief feedback questionnaire (response rate: 71.6%). Aggregated responses indicate a moderately positive impact of the videos on viewer attitudes. As seen in Table 4, the most frequent response for the three items that were normally coded (items 1, 3, 5) was ‘agree’. Similarly, the most frequent response for the two items that were reverse coded (items 2, 4) was ‘disagree’.

**Table 4:** Summary and aggregated results of brief feedback questionnaire responses (*n* = 1104)

Item	Mean	Median	Mode	SD	Range
1. The video changed my attitudes towards people with severe mental illness for the better	3.94	4	4	0.76	1–5
2. My understanding of stigma has not increased after watching this video	2.53	2	2	1.07	1–5
3. The video led me to better understand recovery from severe mental illness	3.90	4	4	0.8	1–5
4. The video had little impact on my outlook towards people with severe mental illness	2.10	2	2	0.99	1–5
5. My behaviour to people with severe mental illness will change for the better in light of this video	4.03	4	4	0.77	1–5

*SD* standard deviation

As stated, the questionnaire also asked viewers to add basic demographic information about themselves; namely age, sex and profession. These were aggregated to assess for overall demographic patterns in responders, and can be seen below in Table 5.

**Table 5:** Basic demographics of viewers who completed questionnaires (*n* = 1104)

Demographics	Total number^a^
% female	72.2
Age in years, mean (SD, range)	34 (14.7, 14–80)
Age in years, median (IQR)	29 (21–44)
Age in years, n (%)	
< 18	11 (1.0)
18–30	555 (51.8)
31–49	274 (25.6)
50+	232 (21.6)
Profession, n (%)	
Student	472 (45.5)
Social service	188 (18.1)
Health care	152 (14.7)
Business/ admin	62 (6.0)
Arts/ culture	50 (4.8)
Other	57 (5.5)
Retired	36 (3.5)
Unemployed	20 (1.9)

*SD* standard deviation

^a^ Numbers do not always add up to 1104 because of occasional missing values due to non-response. We did not impute new values in place of missing values given the small numbers involved. Also, this table is purely descriptive and the sub-groups are not used as a basis for inferential analysis

As said, we conducted 6 focus groups (*N* = 30) to further assess impressions of the videos and subjective impact, allowing us to triangulate the questionnaire and focus group data. Of the 30 participants, a total of 22 were under the age of 35 (73%) and 20 (67%) were women.

The focus group data was highly-consistent with the questionnaire data, indicating that the videos left positive impressions on viewers. Four clear themes emerged from the analysis of focus group data, indicating the core factors which led to these positive impressions and positive subjective impact, these being: (i) educational and informative; (ii) real and relatable; (iii) attention-grabbing; and (iv) change-inducing, which are dealt with in turn below.

### Educational and informative

Focus group participants consistently stated that they found the videos educational and informative. This was less about facts and figures, but more about illustrating the day-to-day realities of life with a mental illness. Indeed, it was frequently stated that the videos made an ideal introduction to issues and challenges associated with living with a mental illness.

For example, one participant stated that the videos “definitely start a dialogue, especially among people that really don’t know anything about mental illness, and it could just be a very good first introduction”. Another participant stated that “these are things that a lot of people in mental health circles talk about all the time. But, the average person on the street doesn’t know about”, with another simply concluding that “I think it’s a great educational tool”.

A number of videos focused on life with a specific disorder. Participants regularly stated that these videos were particularly informative. For instance, one participant who watched the video ‘Faces of Borderline Personality Disorder’ stated: “I didn’t really know anything about borderline before watching that video. And when you watch that, you learn more”. Likewise, one participant attended a screening which included two videos about schizophrenia, stating that:

I enjoyed the fact that two of them were on schizophrenia … I liked that because … I know a lot more about like anxiety and depression; just the ones that people are more comfortable talking about. So I think it was interesting to …understand the specific barriers they’re facing, which are even more I guess extreme …

Indeed, many participants noted that the videos were especially informative about the social challenges faced by people with mental illness, as well as the actions they took to facilitate recovery. For example, two popular videos focused on the recovery of a single individual using the digital storytelling method (“Jennifer’s Story” and “Audley Inspirational”). One participant stated she found these videos ‘really cool’ because they animated the recovery ‘journey’ by discussing the barriers and facilitators to recovery:

With Jennifer, um I liked that she told her story…how she became homeless and how she had to find a group home and the application process…it almost gives me a day-by-day, what she had to do to get to where she is now, which I thought was really cool. And I think Audley, he gave us kind of a journey, also, because he gave specific advice which was exercising and finding a project and stuff like that. So, I thought that was pretty useful information.

Of note, some focus group participants were clinical students or health care providers. A common sentiment expressed by these individuals was that the videos supplemented their more abstract ‘book knowledge’ with enlightening stories grounded in the real world. For example, one participant attended a screening for pharmacology students, stating that:

I observed, just watching it because I’m at the back, and I’m watching everybody watch these videos and hearing the questions that they’re asking. They felt that this was very valuable and even helping them in their profession to becoming pharmacists. They’re like ‘oh, this makes sense why this is important’, and this is actually like, reaffirming why we have to pay attention to interactions and why we need to be more personal and be more present with our clients.

### Real and relatable

Focus group participants frequently used words such as ‘relatable’ and ‘real’ when describing the videos. Most commonly, this was discussed in regards to the presentation of real-world individuals and their stories, which were constantly praised for their ability to ‘humanize’ and ‘personalize’ mental illness. For instance, one participant stated that:

It’s so great to actually like hear someone’s story and put a face; and like feel that connection…what they go through in our world; and their struggles; and how they surpass them; and how it’s made them stronger; and the services that were there to help them, or the lack thereof.

Indeed, this focus on the recovery of local individuals within the videos was considered a very powerful and effective trope by the focus group participants. One participant mentioned that “actually showing a person” was more powerful for them than “just [mental illness] being the central theme and discussion of the video”.

Other participants praised the videos for the multi-dimensional in-depth portrayal of people with mental illness, with one stating that “what worked is we actually got to see her as a person. We got to see her life, her transitions, her talents, you know, her relationships, and that’s what drove it home”. A similar sentiment was expressed by a student participant who stated that “you kind of learn about these concepts in more of an abstract form, and you rarely get to see how it affects someone… but the videos just give you a very different perspective and makes it more human”.

Others noted that a strength of the videos was their well-rounded and normative portrayal of people, moving beyond the mental illness label to show individuals as active citizens. For example, one participant declared that one video about a man in recovery was “Marvelous! Just it shows him as a human being, it shows him as a brilliant artist. It shows him as a member of a community; it shows him as a citizen, you know? It was just wonderful”.

Indeed, participants regularly praised the focus on local ordinary people and the depiction of people with mental illness in a multi-faceted positive manner. As one participant aptly stated, “I like these videos because they use ordinary people – I can’t identify with a multimillionaire, but I can identify with someone who is like me”. Another participant noted approvingly that “the videos were made by someone with their own experiences…I just retain it better…it impacts you more strongly than if it’s some like fictional thing or an abstract article”.

Similarly, participants noted that the videos often helped narrow social distance, with one mental health worker stating that “these videos, the ways that they kind of get into the personal lives of those people, it really bridges a distance”. Another participant stated:

You can relate…as everyone comes to forks in the road in their life. And I’ve had lucky breaks but if I would have had an unlucky break you look at these people and you think ‘God, that could’ve been me’.

This view is encapsulated by a participant whose brother had schizophrenia, who noted that the videos were educational, relatable and thought-provoking all in one. This participant talked about a screening thus:

I think it was amazing…this impression that there’s no road to recovery is really the worst part of the stigma…So, I, I find that – I found that part incredibly relatable, as my brother has improved throughout his life…And to be honest, there’s been points in my life where I wondered how close I could have been to being in a shelter…I thought they were all very relatable.

### Attention-grabbing

Focus group participants regularly reported that the screenings grabbed their attention and left a memorable impression, leading them to further engage with the videos and their content. For example, one said that the videos were “very good…It had my attention the whole time. And it made me want to watch more of them. So I think you hit the ball out of the park on that one!”.

Relatedly, participants often noted that the videos were produced with a high level of skill and professionalism, with one participant stating that they “were excellent…like industry standard, you know. It looked like professional, you never would’ve guessed that these were people who 12 months before hardly knew anything about cameras or videos”. Indeed, some focus group participants had experience in videography, and they were particularly impressed, with one stating that:

I really liked that video because it just was so well done from like a production point of view. You can tell they had a storyboard, and, you know, they obviously – they shot out of order, and they had different sequences acted out. And, you know, the editing was – Like there wasn’t any point where I felt like the editing has stopped me from the story. I think it was just really well done…

Participants also highlighted the novelty value of the bottom-up emphasis on lived experience of mental illness, which offered unique (and under-reported) perspectives and storylines. In fact, many participants enjoyed the films and found them attention-grabbing specifically for these reasons. For example, one participant described their experience as such:

It is powerful to put the camera and the storytelling in the hands of the person who is, who has lived the experience of mental health…seeing someone – hearing a story from the perspective of someone who has, you know, lived this life and is able to tell it – tell it cleanly and really – really well, in that way, is just – I mean, there’s nothing else like it.

Likewise, some participants reflected positively on the granular-level technicalities of the videos; including discussion of animation, accompanying music and filming locations. For example, one film, ‘Off-Suit’, included extensive animation. One participant found this memorable, stating that “I found I haven’t seen very much [animation] recently and just to sort of have it stick in my head as like a different visual. That was really, really nice.” Another participant stated “some of that music, I can still hear…and I mean the settings that they chose to show themselves in as well and how they moved within those settings, like their body language…It was really rich”.

In short, many participants perceived the videos as high-quality, gripping productions; this allowed them to engage fully with the core issues under discussion such as stigma and recovery. This subjective experience is neatly encapsulated in the quote below:

I think [the videos] are good…I thought they were amazing…I was impressed by the level of craft in the videos. They were very watchable, and um engaging…And I think that the films are like a really interesting way of letting people show exactly what [mental illness] means in reality. Like we talk about this idea of stigma and mental health and how like, I don’t know, It ends up being like kind of theoretical…until you’re faced with a human being that has to deal with those stigmas and all those labels. And the way that they manifest in reality is so different from how you ever read about it

### Change-inducing

As previously discussed, the videos were frequently considered educational and informative. Moreover, participants reported that they contained relatable characters, and that the production was professional and attention-grabbing. All this interacted, leading to reported changes in understandings, attitudes and even behaviours. For example, one participant described how her own levels of stigma towards people with mental illness decreased after watching the videos:

I think having the videos is your best opportunity to being able to reduce stigma with other people because I know, like I said, watching it myself it brought me down to the level of being like “Okay. This is like a real human thing that you actually – that people go through, right?”. I think there’s a perception that people with mental illness, there’s no – they’re never gonna get better, they’re just, they’re not trying to work on it…but from my perception, I can see how people work on it. And so for me, the stigma went down a little bit watching some of the videos and seeing that…it reduced my judgment mindset.

Similarly, some participants described how the videos led them to reframe their perspectives about people with mental illness, which led to increased empathy and understandings. For example, one participant stated that the videos were enlightening as they showed people “in a recovery path. So, you could see that while you’re not totally cured…you’re able to function in society and, you know, be just like everyone else”. Another stated that her experience at a screening “increases your empathy and makes you realize all of these things you don’t think about”, while another participant described how it made them realize that people with mental illness “are suffering…it’s affecting their brain or something else. But they’re not bad people. In fact, they’re artists, and they’re, you know, doctors, lawyers, engineers, whatever else”.

Notably, some participants implied that the videos changed their behaviours towards people with mental illness for the better. This was often related to the development of empathy, which could prompt kinder and more engaging behaviours. For example, one participant discussed their experience viewing a video entitled “Psychotropic Side Effects 101”, stating that:

I teach at university, so one of the videos about psychiatric medication side effects, said that people get drowsy, you know. It doesn’t mean they’ve stayed up all night partying or, or that people yawn, sometimes fall asleep. So, I see that happen in class and after watching that video, uh, I would say to myself ‘that poor person probably has like mental illness, and they’re on psychiatric medication and they’d much rather be sitting listening to me teach, but the medication is so strong’. Whereas before I watched that video I’d be more like ‘ugh’ and think like ‘God, how dare they sleep when I’m talking’ (laughs). So, at a practical level the videos have actually changed some of my empathy.

Others made similar remarks, which were often centered around the wider theme that the videos ‘humanized’ or ‘personalized’ mental illness and its associated challenges, leading to changes in empathy and understanding. For example, one participant noted the positive change-inducing value of these videos, which contained hopeful stories of recovery, in contrast to Hollywood-type violent dramas which commonly induce fear by misrepresenting schizophrenia:

I think showing shorts [short videos]…I think it is good…I think it is actually like good clickbait because like you watch it, and you’re like, “Oh! This is actually like really lovely and humanizing, and I no longer fear people with schizophrenia or whatever.” You know? Hopefully.

To close the results section, we would like to note that the researchers did not independently identify any other major themes that are not integrated into the results section above. That said, we did note a very small amount of less positive comments. A few people stated that some videos were too long thereby making them less effective, preferring the shorter videos. Also, a few participants stated that the videos could have included more documentary-style facts or statistics. However all researchers agreed that this did not constitute a theme, as the less-positive comments were very rare, and in fact were completely absent from some of the focus groups.

## Discussion

There are two key findings to this study. Firstly, the results indicate that participatory video with people with mental illness is a feasible anti-stigma intervention. Secondly, the data suggests that the resultant videos and organized screenings positively affect viewers and could be an effective means of stigma reduction.

These results have policy implications. As stated, stigma reduction has become a key target for mental health policy in various jurisdictions across the world [11, 14]. In Canada, the most-recent ‘Mental Health Strategy for Canada’ states that national priority 1.1 must be to ‘increase awareness and reduce stigma’ [15]. As noted, evidence suggests that contact-based sessions can dispel negative stereotypes and improve attitudes towards people with mental illness, making them an effective anti-stigma intervention [19–21].

Importantly, the PV screenings detailed in this study integrated many of the recommended evidence-based critical ingredients of contact-based sessions. For example, Chen et al. [17] note that ‘engaging contact reduces stigma’, and state the importance of ‘speakers, message and interaction’. Similarly, Knaak & Patten [18] recommend that effective contact-based sessions should ‘include personal testimony’, ‘dispel myths’ and ‘emphasize and demonstrate recovery’.

At the PV screenings, at least two members of the workgroup participated in the panel discussion and question period, answering questions and interacting with audience members. Likewise, the videos frequently presented stories of personal recovery from a first-person perspective, which was re-emphasized in the panel discussion. This gave a clear and hopeful message that viewers found educational and informative, dispelling myths and stereotypes in the process. As witnessed in the results, this also increased viewer empathy, which has been identified as a powerful mediator of prejudice reduction in a seminal meta-analysis [40].

Notably, the adopted PV approach also contained important additional elements which are not necessarily captured in a traditional contact-based session. For example, many focus group participants expressed surprise and amazement at the high-quality of the videos. By the same token, the videos frequently went ‘behind the scenes’ to show people with SMI competently performing everyday activities in their workplace, homes or community spaces. All this could further help dispel myths and disabuse viewers of stereotypical notions that people with SMI are violent, incurable or inept [28]. In short, PV gives added value to contact-based sessions as it intensely enacts the ‘seeing is believing’ mantra, illustrating in a multi-faceted manner the strengths, capabilities and competencies of people with SMI.

Similarly, videos produced by PV programs have a wider potential reach than contact-based sessions. This could be especially important in large countries such as Canada and Australia where significant portions of the population live in rural and remote regions, often with specific mental health challenges, including stigma [41]. This remoteness could mean that contact-based sessions with local people are not always practical or ethical, due to cost and privacy issues. As such, bottom-up participatory videos from similar communities may make a viable and economic alternative. Indeed, recent (non stigma) research suggests these methods could be feasible and effective with groups such as rural Aboriginal youth [24, 42]. Likewise, a recent study in rural Nepal successfully used similar visual methods (photovoice) as the basis for an organized anti-stigma intervention targeted at local primary care workers [43]. This emerging body of research indicates that PV has the potential to reduce stigma in diverse locations, and could be an important tool in the effort to improve global mental health.

### The RADAR model

Given this experience, we have packaged the process into a discreet intervention that we call the RADAR model, which can be implemented by people with mental illness elsewhere. To assist others with implementation, we are creating a short toolkit and manual in lay language, which will shortly be available at the project website [36]. Such implementation tools are important as a recent review of anti-stigma interventions noted ‘the poor quality of the interventions, which were sometimes delivered without training, manualisation or fidelity checks’ [21].

The RADAR model has five key components. Firstly, an action-plan should be developed in a bottom-up manner with feasible deadlines and targets. Secondly, workgroups must be provided with appropriate video equipment and training in both camera work as well as analytical thinking. Thirdly, workgroups should be supported by a facilitator with videography skills during the production and editing phase. Fourthly, participant-led screenings should be organized that include an introduction, video screenings, question period and panel discussion. Fifthly, videos should be disseminated on dedicated social media channels and websites, then shared with relevant organizations and local media.

Of note, the study points to three critical ingredients that make videos effective in reducing stigma. First, videos should aim to educate and inform, shedding light on underlying issues such as stigma, recovery and social challenges associated with mental illness. Second, videos should be grounded in the realities of everyday life with mental illness, using relatable local people in recovery as mainstays of the videos. Third, videos must be attention-grabbing and engaging through high-quality production. All this may ultimately induce viewer change.

Importantly, the feasibility targets for the number of screenings to the general public were not met at two sites. In fact, it proved considerably easier to organize screenings within our existing networks, mainly by taking slots at pre-existing routine events, such as a class in a university course or a local hospital grand round. This indicates that dissemination may be most successful where workgroups exploit existing networks and piggy-back on pre-existing routine events.

Indeed, the workgroups opportunistically organized several screenings to mental health service user groups and organizations as word of the project spread through local networks. This deviation from the action-plan was considered a sign of success, as the workgroups deployed agency and initiative by organizing screenings to an important audience. It is also consistent with the wider PV approach, which demands participant control over dissemination [23, 25, 27].

### Limitations of the study and future research

There are a number of limitations to the present study. First, the quantitative measure of impact was purpose-built and relatively simple, and was not administered before the screening. This approach only gives limited information about the effect of video viewing on external stigma. However, this project was primarily feasibility research of an understudied intervention in an understudied population, meaning that the gathering of preliminary evidence for impact is warranted. This was also a preference of the participants during co-design. Future research should test more formally the impact of such videos longitudinally through repeated measures with validated instruments.

Second, we collected and analyzed viewer data generically, without stratification, in order to assess general impact. For example, each focus group included a heterogenous mix of viewers- including students, health care providers, mental health service users and the general public. Homogenous focus groups of each category may have given different results, however this proved impractical due to the tight schedules of potential participants. By the same token, we did not stratify the quantitative feedback from the video screenings by age, gender or other variables. This was not an a priori aim of the study, and such post-hoc analysis may risk inferential error due to issues of sample size, power and multiple testing [44]. Similarly, this quantitative data was derived from a simple purpose-built questionnaire rather than a well-validated stigma questionnaire, meaning that it is more suitable as a general indicator of impact rather than a basis for nuanced sub-group analysis. However, future research could use formal experimental methods to assess the impact of video viewing on different sub-groups using validated measures.

Third, there is a possibility of bias in various aspects of the study. For example, selection bias may have affected screening attendance, as these were arranged opportunistically at sympathetic venues (e.g. hospitals, universities and service-user organizations) and likely included people who were already receptive to mental health issues. Similarly, there is a possibility of response bias, especially in the focus groups, as participants may have given socially desirable answers given the presence of other people in the group- some of whom introduced themselves as mental health advocates or people with mental illness. This could have inhibited the expression of less positive comments, which may have emerged through a more private methodology such as in-depth interviews. Moreover, it should be noted that questionnaire and focus group respondents were mostly young female students, with a minority of men and older adults. This sampling bias places some limits on generalizability. Future research should purposely test the impact of PV on groups who are traditionally hard to reach in stigma research, such as men and older adults.

Fourth, we did not formally measure variables such as level of functioning, specific diagnosis or educational background, as this was considered inconsistent with our inclusive recovery-oriented approach during co-design. This may limit the generalizability of the results to other groups of people with mental illness. That said, all participants regularly attended a psychosocial rehabilitation centre and self-identified as in recovery from a SMI. Moreover, the content of the resultant videos indicates that participants had experienced a range of serious issues including homelessness, hospitalization and unemployment [28]. This means that many participants would conventionally be considered as being at the more severe end of the spectrum. Thus, the success of the project implies that PV is recommended and suitable for all people in recovery from SMI. This is especially the case given that PV is always a group effort characterized by peer support and differing roles for workgroup members according to strengths and capabilities [25, 27].

Fifth, this paper focuses on the impact on viewers, and does not report the impact on the workgroup members with SMI who actually made the videos. Theoretically, involvement in the process may have led to a reduction in self-stigma and an increase in empowerment among workgroup members [5, 20, 22]. As such, the research team recently completed end-of-project qualitative interviews with workgroup members to explore the subjective impact of participation in the program. These data will form the basis for another research paper, allowing us to assess if involvement in a PV project can reduce self-stigma.

## Conclusions

To our knowledge, this study is the first large-scale multi-site project examining the feasibility and impact of a participatory video program for people with mental illness. The results indicate that PV is a feasible method, as participants with mental illness exceeded the vast majority of targets in terms of project participation, video production and screening organization. The questionnaire and focus group data give preliminary evidence that these videos can reduce stigma in viewers. As such, participatory video should be considered a promising practice in the ongoing struggle to raise awareness and reduce stigma.

## Data Availability

The excel datasets analysed during the current study are available from the corresponding author on reasonable request.

## Abbreviations

MHCC: Mental Health Commission of Canada
SMI: Severe mental illness
